# Performance of islets of Langerhans conformally coated via an emulsion cross-linking method in diabetic rodents and nonhuman primates

**DOI:** 10.1126/sciadv.abm3145

**Published:** 2022-06-29

**Authors:** Aaron A. Stock, Grisell C. Gonzalez, Sophia I. Pete, Teresa De Toni, Dora M. Berman, Alexander Rabassa, Waldo Diaz, James C. Geary, Melissa Willman, Joy M. Jackson, Noa H. DeHaseth, Noel M. Ziebarth, Anthony R. Hogan, Camillo Ricordi, Norma S. Kenyon, Alice A. Tomei

**Affiliations:** 1 Diabetes Research Institute, University of Miami Miller School of Medicine, Miami, FL 33136, USA.; 2 Department of Biomedical Engineering, University of Miami, Miami, FL 33146, USA.; 3 Department of Surgery, University of Miami Miller School of Medicine, Miami, FL 33136, USA.; 4 Department of Microbiology and Immunology, University of Miami Miller School of Medicine, Miami, FL 33136, USA.

## Abstract

Polyethylene glycol (PEG)–based conformal coating (CC) encapsulation of transplanted islets is a promising β cell replacement therapy for the treatment of type 1 diabetes without chronic immunosuppression because it minimizes capsule thickness, graft volume, and insulin secretion delay. However, we show here that our original CC method, the direct method, requiring exposure of islets to low pH levels and inclusion of viscosity enhancers during coating, severely affected the viability, scalability, and biocompatibility of CC islets in nonhuman primate preclinical models of type 1 diabetes. We therefore developed and validated in vitro and in vivo, in several small- and large-animal models of type 1 diabetes, an augmented CC method—emulsion method—that achieves hydrogel CCs around islets at physiological pH for improved cytocompatibility, with PEG hydrogels for increased biocompatibility and with fivefold increase in encapsulation throughput for enhanced scalability.

## Teaser

Islets conformally coated by emulsion method with PEG hydrogels show improved performance.

## INTRODUCTION

Insulin-secreting β cells are selectively destroyed in autoimmune type 1 diabetes (T1D) (*1*), rendering the body unable to maintain glucose homeostasis. Insulin replacement therapy has been used for nearly a century (*2*), although technology has taken the practice from simple injections to glucose-sensing, insulin-dispensing pumps controlled sophisticated algorithms to mimic natural insulin secretion (*3*). While these therapies have substantially improved metabolic control, secondary T1D complications, and quality of life for patients with T1D, their performance is still suboptimal as compared to native biological glucose homeostasis maintained by β cells.

β Cell replacement through transplantation with chronic immunosuppression has demonstrated to be safe and more effective in improving metabolic control and quality of life in patients with severe T1D while reducing hypoglycemia unawareness and secondary complications (*4*). Successful transplantation and engraftment of islets could represent a biological cure to T1D, but the deployment of this therapy is mired by challenges (*5*, *6*) associated with allograft rejection (*7*), recurrence of autoimmunity (*8*), and poor engraftment due to inflammation, hypoxia (*9*), and poor revascularization (*10*), which are dependent on the islet transplant site.

Islet encapsulation (*11*) is a family of bioengineering strategies that collectively aim to enclose islets in a semipermeable material so that when transplanted, hormones, nutrients, waste products, and oxygen can freely ingress or egress between the donor islet and the host tissue, while host immune cells are physically blocked from direct contact with the islet. Thus, islet encapsulation may prevent rejection of allogeneic and xenogeneic islets and allow reduction or withdrawal of chronic immunosuppression, increasing the safety (*12*) and efficacy (*13*) of β cell replacement therapies.

Macroencapsulation (*14*) uses a single device to contain the entire transplanted mass of islets; this approach has proven effective in small-animal models, but the scale-up to large animals and humans is complicated by the fact that these devices are typically designed to host a monolayer of islets and so they require a large two-dimensional (2D) area. Microencapsulation (*15*) encases individual islets in a hydrogel microdroplet that is typically fixed in size at 500 to 1000 μm in diameter: For curative doses of islets in humans, this results in an overall graft volume on the order of liters (*16*). Such a volume can only practically fit in the intraperitoneal cavity, a site with poor oxygen tension, poor graft survival, delayed glucose sensing and glucose-regulated insulin secretion, and that does not permit graft retrieval.

We have previously reported on a fluidic method (*17*) to encapsulate islets in a hydrogel capsule that is a few tens of micrometers in thickness independently from the size of islets (*16*) (the coatings conform to the islet shape and size), which results in an overall graft volume on the order of milliliters for a human. Minimal capsule thickness and graft volume confer critical advantages to conformal coated (CC) islets compared to other microencapsulation platforms: More facile diffusion of nutrients, glucose, and insulin allows physiological glucose-stimulated insulin secretion (GSIS) by CC islets with no insulin delays, while smaller graft volumes comparable to those of naked islets allow transplantation of CC islets in confined well-vascularized sites and are not limited to the avascular and nonretrievable peritoneal cavity, thereby improving metabolic control of CC graft recipients. Conformally coated murine (*18*) and human primary and stem cell–derived islets (*19*) showed efficacy in diabetes reversal in mice, even in immunocompetent allogenic mice without immunosuppression. However, the coating process required the hydrogel coating precursor polymer (i) to be maintained at an acidic pH of 3.5, exposing islets to nonphysiological pH levels, and (ii) to be run through the encapsulation device before gelation occurred in approximately 4 min, allowing no more than 2000 islet equivalent (IEQ) to be coated per run and reducing the process efficiency, and (iii) required the use of a viscosity-enhancing additive to polyethylene glycol (PEG) hydrogels, which is likely to reduce PEG-based coating biocompatibility. Here, we report testing of the CC platform in a nonhuman primate (NHP) model of T1D, a first step in the translation of the technology to humans, and identification of important limitations of the technology in terms of cytocompatibility, biocompatibility, and scalability in large-animal models that were not observed previously in small-animal models of T1D. Then, we present the design and validation of a modified CC platform that is conducted entirely at neutral pH and does not require any viscosity enhancers, thereby generating pure PEG hydrogel coatings with higher coating process throughput than the previous direct method (DM). The performance of polystyrene beads and islets conformally coated with this emulsion method (EM) was tested in vitro and in vivo in several clinically applicable transplant sites in small- and large-animal models of T1D.

## RESULTS

### DM CC impairs functionality of primary NHP islets and results in poor biocompatibility in the omental pouch of diabetic NHPs

#### Effect of NHP islets with DM CC in vitro and in vivo in diabetic NSG mice

**Fig. 1.**
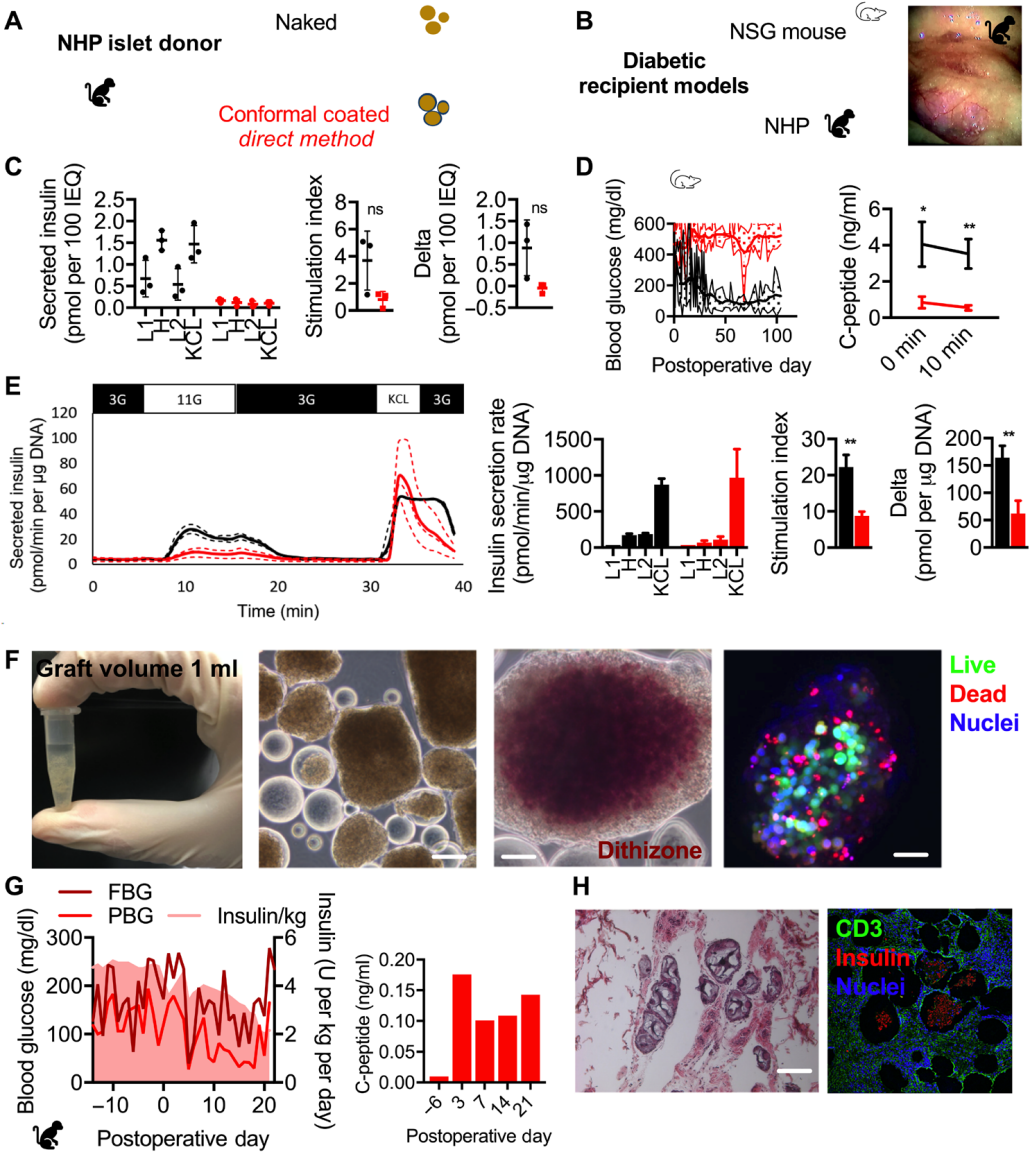
In vitro and in vivo characterization of NHP islets CC by DM. (**A**) Schematic of NHP islet donor and naked (black) and CC DM (red) islets. (**B**) Schematic of diabetic recipient model NSG mouse and NHP (left) and a photograph of NHP omentum with CC DM NHP islets obtained via laparoscopic camera. (**C**) GSIS of naked (black) and CC DM (red) NHP (*n* = 3 biological repeats from distinct donors, *n* = 3 technical repeats per donor). (**D**) Daily blood glucose readings of mice receiving 2000 to 4000 IEQ of naked (black, *n* = 5) and CC DM (red, *n* = 10) NHP islets and stimulated C-peptide measurement (*n* = 3 for both conditions). *P < 0.05 and **P < 0.01. (**E**) Perifusion (dynamic GSIS) of naked (red, *n* = 3 technical repeats) and CC DM (red, *n* = 3 technical repeats) NHP islets pooled from two donors (3G, 3 mM glucose; 11G, 11 mM glucose; KCl, 3 mM glucose and 30 mM KCl). Technical repeats are presented as dotted lines, and means are presented as solid lines. (**F**) Photograph of a 1.5-ml microcentrifuge tube containing 30,000 IEQ (1-ml tissue graft volume) of CC DM NHP islets destined for transplant in omental pouch of a diabetic NHP recipient (leftmost); phase-contrast image of CC DM NHP islets (middle left; scale bar, 200 μm); phase-contrast image of dithizone-stained CC DM NHP islets (middle right; scale bar, 200 μm); confocal micrograph of a CC DM NHP islet [rightmost; live cell cytoplasm (green), dead cell nuclei (red), all nuclei (blue); scale bar, 200 μm]. (**G**) Metabolic data for diabetic NHP recipient H15C35 including FBG (dark red) and PBG (red) and daily EIRs (pink, shaded) in the left graph and fasting C-peptide in the right graph. (**H**) Light microscopy images of hematoxylin and eosin (H&E)–stained (left) and immunofluorescence confocal micrographs (CD3 in green, insulin in red, nuclei in blue) of explanted omental tissue containing islet grafts (scale bars, 200 μm). ns, not significant.

With a view toward translating CC islet therapy to treatment of T1D in humans, islets were isolated from three NHP donors and CC with the DM (Fig. 1A) using PEG and a viscosity-enhancing peptide (VEP) additive as we previously reported (*19*). GSIS assay revealed that while naked (unencapsulated) islets exhibited a typical GSIS profile (Fig. 1C), the performance of DM CC NHP islets was notably diminished in terms of absolute quantities of insulin (Fig. 1C, left), stimulation index (H divided by L1; *P* = 0.09; Fig. 1C, middle), and delta (H minus L1; *P* = 0.07; Fig. 1C, right), although the latter two metrics were not statistically significant. DM CC NHP islets did not durably reverse diabetes after transplantation in the fat pad of diabetic NSG mice (Fig. 1, B and D, left), while naked islets did so for up to 100 days with a median reversal time of 14 days. Both fasting and stimulated serum NHP C-peptide was higher in mice receiving naked NHP islets than DM CC NHP islets (Fig. 1D, right; *P* = 0.01 and 0.003, respectively), although NHP C-peptide was measurable in the serum of mice receiving DM CC islets, suggesting partial function.

#### Effect of NHP islets with DM CC on a diabetic NHP

NHP islets were isolated from two additional donors, pooled, and DM conformal coated for transplant into the omental pouch of a diabetic NHP recipient (H15C35; Fig. 1, A and B). In vitro, both naked and DM CC NHP islets exhibited typical dynamic GSIS (perifusion) profiles (Fig. 1E, left), although the naked islets had a higher stimulation index (Fig. 1E, right middle; *P* = 0.003) and delta (Fig. 1E, far right; *P* = 0.003). A total of 33,000 IEQ (5000 IEQ/kg) of DM CC NHP islets (Fig. 1F, middle) had an overall graft volume of 1 ml (Fig. 1F, left). Dithizone staining of DM CC NHP islets revealed most of the interior of the islet staining intense red and a thin layer on the exterior not staining red (Fig. 1F, right). Live/dead staining of DM CC NHP islets revealed a very alive interior, with dead cells being present mainly on the islet periphery. The DM CC NHP islets were implanted within a biological scaffold in an omental pouch of a diabetic NHP using laparoscopy. Steroid-free immunosuppression was used.

Posttransplant recovery of the transplanted NHP was abnormal, as the animal did not regain a normal appetite and appeared less active than expected. Figure 1G (left) shows fasting blood glucose (FBG) (dark red) and postprandial blood glucose (PBG) and exogenous insulin requirements (EIRs; pink); Fig. 1G (right) shows the corresponding fasting C-peptide through the experiment. After transplant, glucose control remained erratic, with minimal but higher than pretransplant fasting C-peptide values. The progressive decrease in EIR mirrors the decreased appetite of the animal, i.e., the animal was not eating and, therefore, required less exogenous insulin. There was no fever or sign of infection. As the animal continued to lose weight and stopped eating, we ended the experiment to eliminate distress and determine the underlying cause of dysfunction. Elective necropsy on postoperative day (POD) 22 revealed a surgical hernia in the umbilicus, which is not uncommon in laparoscopic procedures and would explain the anorexia observed in this animal.

Histopathological examination of DM CC NHP allografts in the omental pouch revealed chronic inflammatory responses to the capsules with the presence of foreign body giant cells on most of the capsules (Fig. 1H) and T cell infiltration (Fig. 1I) on grafts between but not within capsules, indicative of both innate and adaptive responses to the coatings while confirming CC immunoisolation.

### Minimally cross-linked viscous PEG coating solution and a polypropylene glycol (PPG)-based cross-linking emulsion permit CC of human islets with PEG hydrogels at neutral pH

**Fig. 2.**
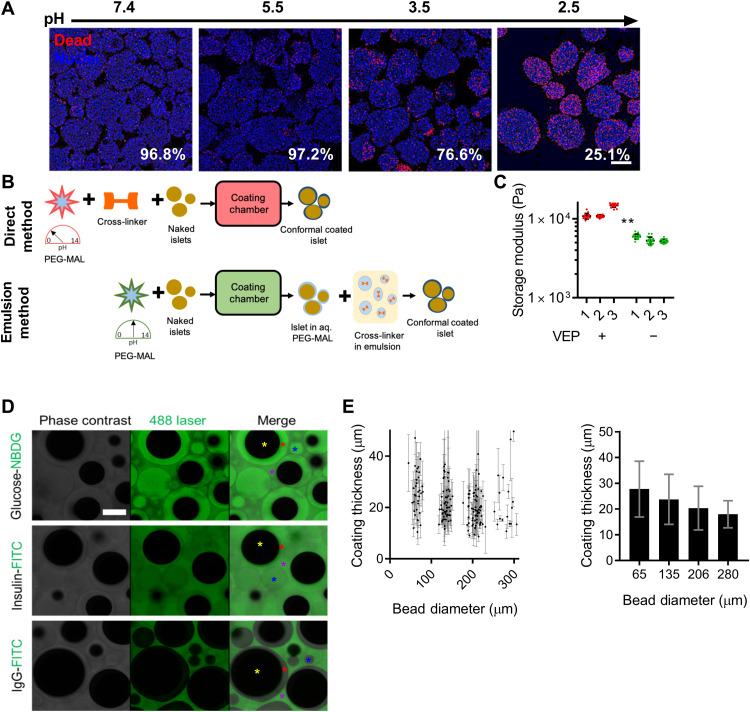
Determining the impact of DM versus EM on islet viability and hydrogel material properties. (**A**) Confocal micrographs of human islets exposed to buffer of pH 7.4, 5.5, 3.5, and 2.5 (all nuclei, blue; dead nuclei, red; scale bars, 200 μm). Viability quantified through image analysis is indicated on the bottom left corner of each image. (**B**) Schematic depicting direct and EM CC processes. (**C**) Material properties (measured as storage modulus) of hydrogels with (red) and without (green) VEP (*n* = 3 technical repeats). **P < 0.01. (**D**) Confocal micrographs (scale bars, 100 μm) depicting diffusion of glucose (top row), insulin (middle row), and IgG (bottom row) into the coating hydrogel. Stars: yellow is polystyrene bead, red is CC, blue is empty hydrogel microsphere, and purple is fluorescent solution. (**E**) Measured CC thickness (bead surface to coating exterior) versus bead diameter (left) and averages by bead size (right).

Live/dead staining revealed that when exposed to HBSS^−/−^ buffer at decreasing pH, the fraction of dead cells on the surface of human islets increased (Fig. 2A). Specifically, the average viability after exposure to pH of 7.4, 5.5, 3.5, and 2.5 was 96.8, 97.2, 76.6, and 25.1%, respectively. Overall, these results indicate that exposure to coating solutions at low pH as performed during DM CC decreases viability and insulin secretion of primary islets. To address these shortcomings of the DM CC method (Fig. 2B, top), we revised the CC process to allow islet coating at neutral pH using a gelling emulsion (EM; Fig. 2B, bottom). Furthermore, we aimed at eliminating the need to include the viscosity enhancer (VEP) to the coating solution, which could have contributed to the high inflammatory responses and suboptimal biocompatibility that we observed in the NHP omentum (Fig. 1H), to generate pure PEG CCs. To that end, we increased the viscosity of the PEG-maleimide (MAL) coating solution to match the optimal viscosity that we obtained with the previously tested additives by partially cross-linking the PEG-MAL with PEG-dithiol cross-linker just before the gelation point as assessed through rheology. Rheological measurement (Fig. 2C) of hydrogel storage modulus revealed that the gels formed with the EM (5479 ± 422 Pa) were softer (*P* = 0.0080) than those of the DM (12155 ± 2319 Pa), but in the same range.

We measured the diffusivity of various molecules of interest in the EM CC hydrogel on polystyrene beads with a diameter of 50 to 250 μm as islet models using fluorescence recovery after photobleaching (FRAP; Table 1). The diffusivity of glucose and insulin was found to be 1110 ± 528 and 187 ± 89 μm^2^ s^−1^, respectively. The diffusivity of immunoglobulin G (IgG) could not be measured because it was lower than the detection limit, implying no meaningful diffusion of IgG across the hydrogel coating. These diffusivity measurements were complemented by confocal micrographs of EM CC polystyrene beads interacting with fluorescently labeled glucose, insulin, and IgG in solution (Fig. 2D). Glucose and insulin were observed to readily permeate the hydrogel coating, while IgG did not, indicative of desired permselectivity for immunoisolation. The Young’s modulus of the EM coating hydrogel surrounding Nit-1 insulinoma cells cluster was measured using atomic force microscopy (AFM) and found to be 2.66 ± 2.35 kPa. The Young’s modulus of the EM coating hydrogel surrounding polystyrene beads was found to be 5.30 ± 1.37 kPa on day 2 after encapsulation and 9.00 ± 7.68 on day 10, indicating sustained stiffness of the coating hydrogel during in vitro culture indicative of coating hydrogel stability.

**Table 1.** Physical properties of EM CC hydrogels.

**Physical property**	**Measured value**
Diffusivity of glucose in coating hydrogel	1110 ± 528 μm^2^ s^−1^
Diffusivity of insulin in coating hydrogel	187 ± 89 μm^2^ s^−1^
Diffusivity of IgG in coating hydrogel	Undefined
Young’s modulus of coating hydrogel on PS beads	5.30 ± 1.37 kPa (day 2) 9.00 ± 7.68 kPa (day 10)
Young’s modulus of coating hydrogel on Nit1 cluster	2.66 ± 2.35 kPa (day 2)

The thickness of the EM CCs was measured across polystyrene beads of four diameters (65, 135, 206, and 280 μm). The coating thicknesses were 28 ± 11, 24 ± 10, 20 ± 8, and 18 ± 5, respectively. It was observed that the coating thickness modestly decreased as the bead diameter increased (65 versus 206, *P* = 0.0008; 65 versus 280, *P* = 0.0022), confirming that overall the coatings were conformal. The coating thicknesses were measured at three locations on the encapsulate beads that were 120° apart. Hence, it was determined that the coating thickness varied, on average, by 27% (mean of relative SDs of three coating thickness measurements) on the same bead.

### EM CC maintains high human and NHP islet viability, encapsulation yield, and GSIS performance

**Fig. 3.**
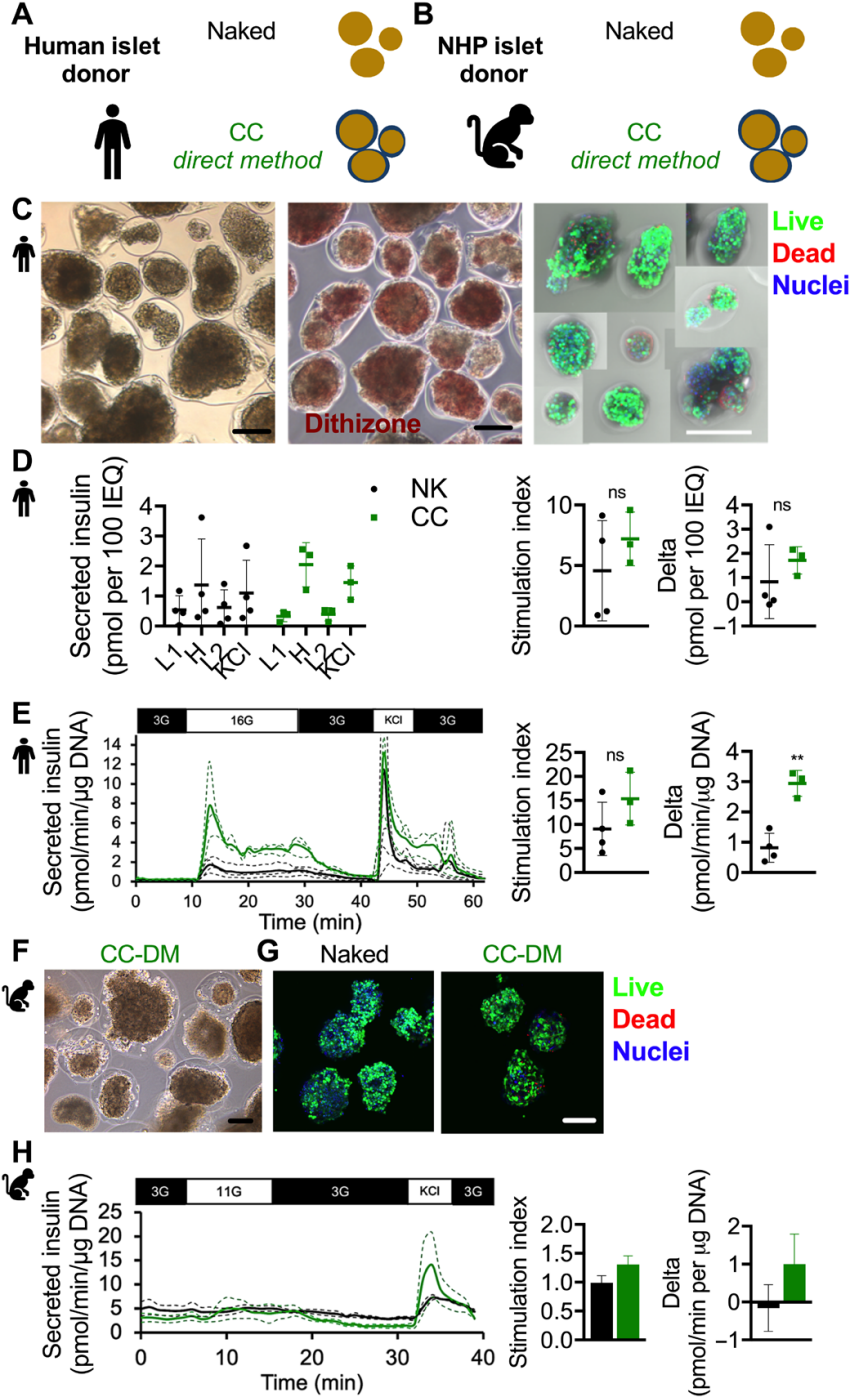
In vitro characterization of EM CC. Schematic of (**A**) human and (**B**) NHP islet donor naked (black) and CC EM (green) islets. (**C**) Phase-contrast image of CC EM human islets (left; scale bar, 200 μm); phase-contrast image of dithizone-stained CC EM human islets (middle; scale bar, 200 μm); confocal micrograph of CC EM human islets [right; live cell cytoplasm (green), dead cell nuclei (red), all nuclei (blue); scale bar, 200 μm]. (**D**) GSIS of naked (black) and CC EM (green) human islets as absolute insulin (L1, 2.8 mM glucose; H, 20 mM glucose; L2, 2.8 mM glucose; KCl, 2.8 mM glucose and 30 mM KCl), stimulation index (H/L1), and delta (H-L1) (*n* = 4 biological repeats from distinct donors, *n* = 3 technical repeats per donor); only three data points for CC EM human islets because islets from two of four donors were pooled. (**E**) Perifusion (dynamic GSIS) of naked (green, *n* = 4 biological repeats) and CC EM (green, *n* = 3 biological repeats) human islets pooled from four donors (3G, 3 mM glucose; 16G, 16 mM glucose; KCl, 3 mM glucose and 30 mM KCl). Biological repeats are presented as dotted lines, and means are presented as solid lines. *P < 0.05 and **P < 0.01. (**F**) Phase-contrast image of CC EM NHP islets (left; scale bar, 200 μm). (**G**) Confocal micrograph of naked (left) and CC EM NHP islets [right; live cell cytoplasm (green), dead cell nuclei (red), all nuclei (blue); scale bar, 200 μm] pooled. (**H**) Perifusion (dynamic GSIS) of naked and CC EM NHP islets pooled from two donors (3G, 3 mM glucose; 11G, 11 mM glucose; KCl, 25 mM KCl). Technical repeats are presented as dotted lines, and means are presented as solid lines. Stimulation index calculated as insulin secretion rate during high step divided by insulin secretion rate during first low step. Delta is calculated as insulin secretion rate during high step minus insulin secretion rate during first low step.

Human islets from four donors (table S1) were conformal coated (encapsulation yield in table S2) using the EM (Fig. 3, A and C, left). The EM (CC EM) permitted the CC process to be conducted on 10,000 IEQ at a time, which represents a fivefold increase in throughput over the DM (CC DM). The yield of the EM was found to be 61.3%, which was not significantly different from that of the DM, which was 57% (*P* = 0.68). The EM CC human islets were observed to stain reddish brown when exposed to dithizone stain (Fig. 3C, middle), and live/dead staining revealed viable EM CC human islets (Fig. 3C, right). Both naked and CC EM human islets exhibited normal static GSIS profiles (Fig. 3D, left) with respective stimulation indices (Fig. 3D, middle) of 4.5 ± 4.1 and 7.2 ± 2.2 (*P* = 0.37) and delta (Fig. 3D, right) of 0.83 ± 1.52 and 1.72 ± 0.57 pmol of insulin per 100 IEQ (*P* = 0.39). The dynamic GSIS (perifusion) profile of both naked and EM CC human islets was typical (Fig. 3E, left), including a first phase peaking and a second phase plateauing of insulin secretion during the dynamic high-glucose stimulation. The perifusion stimulation index was 9.1 ± 5.5 for naked islets and 15.4 ± 5.5 for EM CC islets (Fig. 3E, middle; *P* = 0.19). The perifusion delta was 0.81 ± 0.48 for naked islets and 2.9 ± 0.43 pmol/min per microgram of DNA (Fig. 3E, right; *P* = 0.002).

Next, we tested the EM CC method on NHP islets (Fig. 3B). CC EM NHP islets showed positive dithizone staining (Fig. 3F) and comparable viability (Fig. 3G) and dynamic GSIS (Fig. 3H) to naked NHP islets in terms of stimulation index, indicative of improved functionality compared to the DM-coated NHP islets. We concluded that the EM-CC method, unlike the DM CC method, does not impair viability and insulin secretion of CC human and NHP islets.

### The gonadal fat pad is a superior graft site for CC islets as compared to the intramuscular and subcutaneous sites

**Fig. 4.**
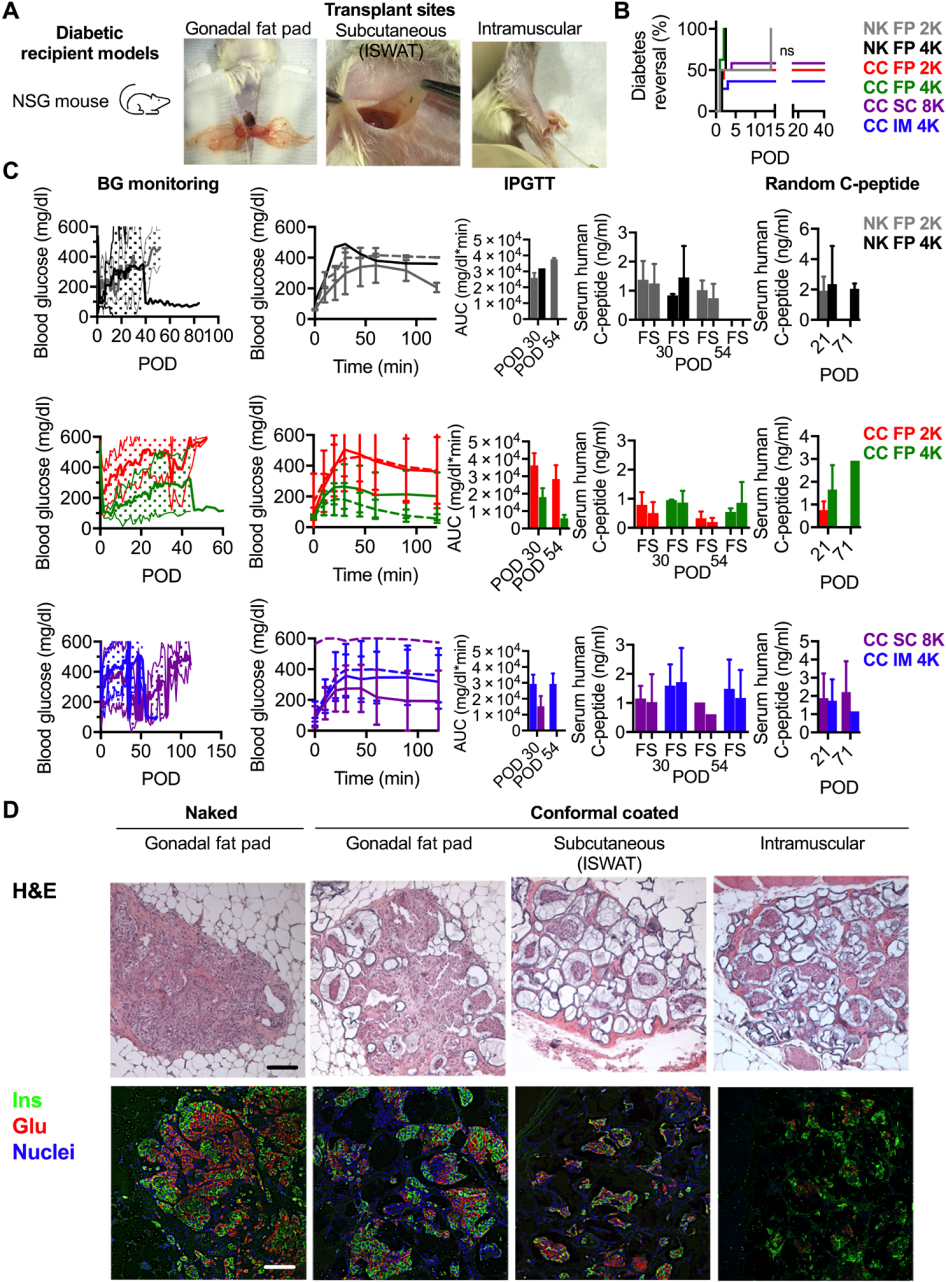
In vivo characterization of EM CC in three sites in immunocompromised NSG mouse model. (**A**) Schematic of diabetic recipient model NSG mouse receiving naked (black) and CC EM (green) human islets and photographs of three transplant sites: gonadal fat pad, subcutaneous (inguinal subcutaneous white adipose tissue), and intramuscular. (**B**) Survival curve depicting diabetes reversal in NSG mice: 2000 IEQ (gray, *n* = 2) and 4000 IEQ (black, *n* = 2) of naked human islets in the gonadal fat pad, 2000 IEQ (red, *n* = 10) and 4000 IEQ (green, *n* = 8) of EM CC human islets in the fat pad, 4000 IEQ of EM CC human islets in the intramuscular site (IM; blue, *n* = 11), and 8000 IEQ of EM CC human islets in the subcutaneous site (SC; purple, *n* = 10). (**C**) Daily blood glucose (left column of graphs), IPGTT blood glucose profile (POD 30, solid lines; POD 54, dashed lines; middle left column of graphs), IPGTT area under the curve (AUC) (middle column of graphs), stimulated C-peptide (middle right column of graphs), and random, nonfasting C-peptide (right column of graphs). The same color scheme outlined in (B) applies. (**D**) Light microscopy images of H&E-stained (top row) and immunofluorescence confocal micrographs [bottom row; insulin (Ins) in green, glucagon (GLU) in red, and nuclei in blue] of explanted gonadal fat pad, subcutaneous, and intramuscular tissue containing islet grafts (scale bars, 200 μm).

Next, we assessed the in vivo functionality of EM CC primary islets in three different clinically applicable sites in parallel. EM CC human islets from four donors were transplanted in the gonadal fat pad, subcutaneous space, or intramuscular space of diabetic NSG mice (Fig. 4A). The measured outcomes are summarized in Table 2. The diabetes reversal curves between the several experimental groups were not significantly different (Fig. 4B; log-rank test, *P* = 0.052).

**Table 2.** Site optimization—Measured outcomes of recipient diabetic NSG mice. AUC, area under the curve; n/a, not assessed.

**Site**	**EM CC or NK**	**Dose** **(k IEQ)**	**Median reversal** **time (day)**	**Mean** **postoperative** **glucose (mg/dl)**	**POD 30 IPGTT** **AUC** (mg/dl*min)	**POD 54 IPGTT** **AUC** (mg/dl*min)	**Mean C-peptide** **(ng/ml)**
Fat pad	NK	2	7.5	286 ± 98	25,936 ± 3,155	37,581 ± 766	1.28 ± 0.64
		4	1.5	201 ± 118	31,990	n/a	1.68 ± 1.21
	EM CC	2	21	435 ± 89	36,281 ± 6,995	28,435 ± 8,007	0.56 ± 0.40
		4	1	195 ± 82	18,003 ± 5,218	5,847 ± 1,993	1.14 ± 0.85
Intramuscular	EM CC	4	Undefined	360 ± 142	29,351 ± 5,870	29,431 ± 6,467	1.55 ± 0.99
Subcutaneous		8	2.5	326 ± 117	15,253 ± 6,421	n/a	1.57 ± 1.20

There was no statistically significant difference between naked and EM CC islets in the fat pad at a dose of 2000 IEQ on POD 30 or 54 (*P* = 0.48 and *P* = 0.41, respectively). There was no statistically significant difference between naked and EM CC islets in the fat pad at a dose of 4000 IEQ on POD 30 (*P* = 0.45). There was no statistically significant difference between EM CC islets in the fat pad at a dose of 2000 and 4000 IEQ on POD 30 (*P* = 0.058), but there was a significant difference on POD 54 (*P* = 0.04). There was no statistically significant difference between EM CC islets in the fat pad and the intramuscular space at a dose of 4000 IEQ on POD 30 (*P* = 0.19), but there was a significant difference on POD 54 (*P* = 0.03).

Serum collected just before (fasting) and 30 min (stimulated) into intraperitoneal glucose tolerance test (IPGTT; Fig. 4C) as well as randomly (Fig. 4C) was assessed for human C-peptide. No statistically significant increase in C-peptide between fasting and glucose-stimulated C-peptide was observed for any experimental group except for EM CC islets in the fat pad at a dose of 2000 IEQ (*P* = 0.02).

Hematoxylin and eosin (H&E)–stained images revealed islet grafts with EM CC islets integrated into the host tissue in all three graft sites (Fig. 4D, top row). Immunofluorescence staining revealed grafts with abundant insulin- and glucagon-positive cells in the fat pad and subcutaneous sites and grafts with minimal insulin- and glucagon-positive cells in the intramuscular site (Fig. 4D, bottom row). We concluded that CC EM human islet graft performance in the fat pad is equivalent to naked human islet grafts and superior to their performance in the subcutaneous and intramuscular clinically applicable transplant sites, with the subcutaneous site performing better than the intramuscular site.

### CC human islets reverse diabetes in the immunocompromised NSG mouse and showed minimal function but improved biocompatibility in the bursa omentalis of an NHP

**Fig. 5.**
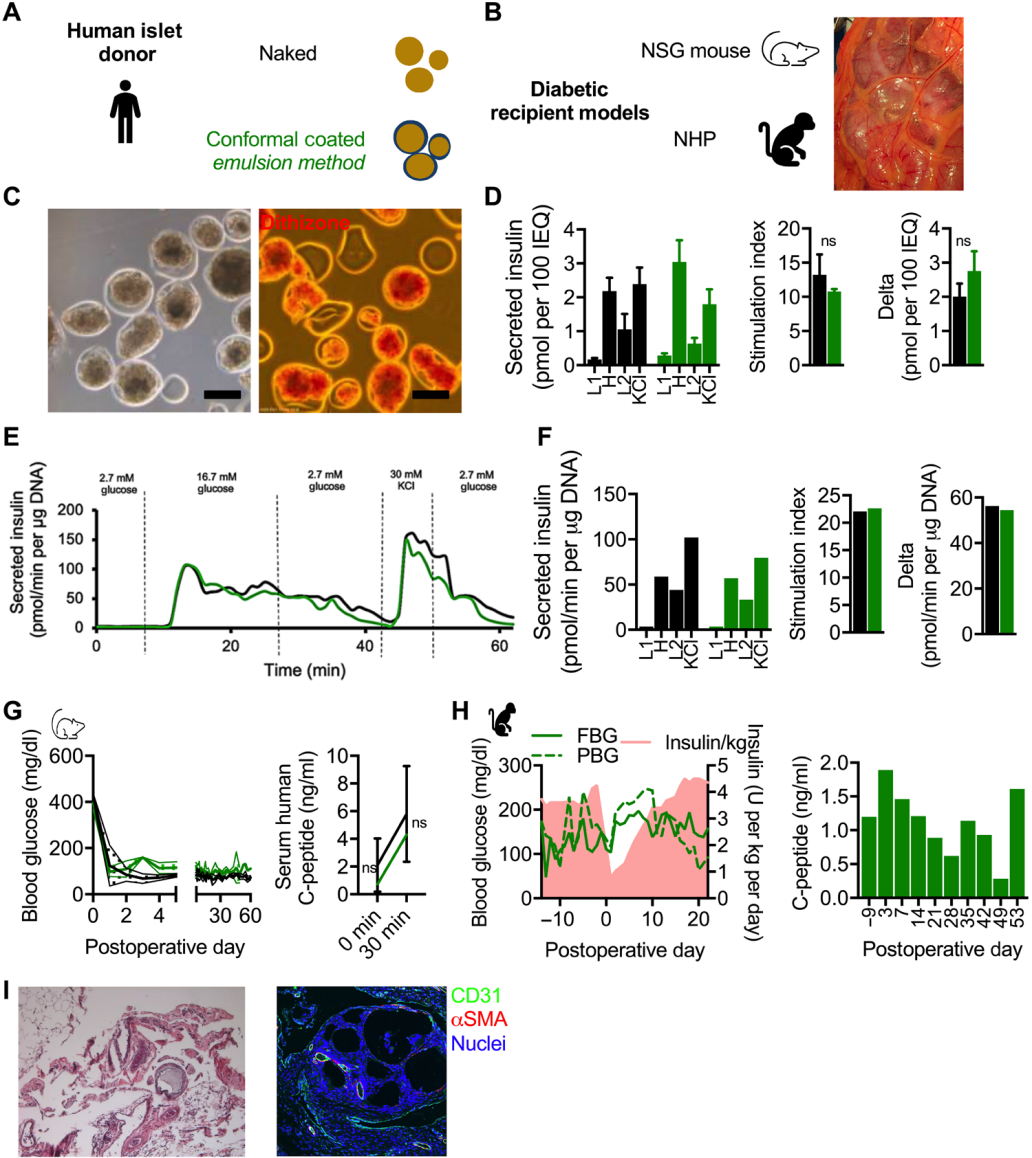
In vivo characterization of EM CC in xenogeneic diabetic mouse and NHP recipient model. (**A**) Schematic of human islet donor and naked (black) and CC EM (green) islets. (**B**) Schematic of diabetic recipient model NSG mouse and NHP (left) and a photograph of NHP omentum with CC EM human islets. (**C**) Phase-contrast image of CC EM human islets (left; scale bar, 200 μm); phase-contrast image of dithizone-stained CC EM human islets (right; scale bar, 200 μm). (**D**) GSIS of naked (black) and CC DM (green) human islets as absolute insulin (L1, 2.8 mM glucose; H, 20 mM glucose; L2, 2.8 mM glucose; KCl, 2.8 mM glucose and 30 mM KCl), stimulation index (H/L1), and delta (H-L1) (*n* = 3 technical repeats). (**E**) Perifusion (dynamic GSIS) of naked (black, *n* = 3 technical repeats) and CC EM (green, *n* = 3 technical repeats) human islets (3G, 3 mM glucose; 11G, 11 mM glucose; KCl, 25 mM KCl). (**F**) Stimulation index calculated as insulin secretion rate during high step divided by insulin secretion rate during first low step. Delta is calculated as insulin secretion rate during high step minus insulin secretion rate during first low step. (**G**) Daily blood glucose readings of mice receiving 4000 IEQ of naked (black, *n* = 3) and CC EM (green, *n* = 3) human islets and stimulated C-peptide measurement (*n* = 3) for both conditions. (**H**) Metabolic data for diabetic NHP recipient H17C39 including fasting (solid green line) and prandial (dashed green line) blood glucose and daily insulin requirements (pink, shaded) in the left graph and serum C-peptide in the right graph. (**I**) Light microscopy images of H&E-stained (left) and immunofluorescence confocal micrographs [CD31 in green, α smooth muscle actin (αSMA) in red, nuclei in blue] of explanted omental tissue containing islet grafts (scale bars, 200 μm).

Human islets from one donor were conformal coated using the EM (Fig. 5, A and C). The EM CC human islets were observed to stain reddish brown when exposed to dithizone stain (Fig. 3C, right). Both naked and CC EM human islets exhibited normal static GSIS profiles (Fig. 5D, left) with respective stimulation indices (Fig. 5D, middle) of 13.2 ± 3.0 and 10.8 ± 0.35 (*P* = 0.23) and delta (Fig. 5D, right) of 2.9 ± 0.38 and 2.8 ± 0.52 pmol of insulin per 100 IEQ (*P* = 0.13). The perifusion profile of both naked and EM CC human islets was typical (Fig. 3E, top), including a first phase peaking and a second phase plateauing of insulin secretion during the high step. The perifusion stimulation index was 22.0 for naked islets and 22.6 for EM CC islets (Fig. 5F, left). The perifusion delta was 0.81 ± 0.48 for naked islets and 2.9 ± 0.43 pmol/min per microgram of DNA (Fig. 5F, right). When transplanted into the fat pad of diabetic NSG mice, both naked and EM CC human islet recipients exhibited a median reversal time of 1 day (*P* > 0.9999) and mean postoperative blood glucose of 88 ± 58 and 110 ± 51 mg/dl (Fig. 5G, left; *P* = 0.48), respectively. C-peptide levels (Fig. 5G, right) increased for both naked and EM CC islet recipients between fasting and 30 min into IPGTT, though not in a statistically significant manner [two-way analysis of variance (ANOVA), row factor, *P* = 0.27].

We transplanted the same batch of EM CC human islets also into a diabetic cynomolgus monkey (H17C59), using a costimulation blockade strategy that was previously demonstrated to be effective for transplantation of encapsulated porcine islets into diabetic NHPs (*20*). On POD 0, the animal received 20,358 IEQ/kg in the bursa omentalis, 1781 IEQ/kg in the pre-peritoneal space, and 1781 IEQ/kg in two mesenteric lymph nodes; EM CC islets were in a suspension in HBSS^−/−^ containing 2% recipient heat-inactivated serum. The animal had some residual islet function before transplant, as evidenced by a fasting and meal-stimulated C-peptide value of 1.2 and 2.2 ng/ml, respectively. However, the main objective of this experiment was to evaluate the biocompatibility of EM CC capsules in NHPs. There was islet function in the early posttransplant period, as evidenced by a fasting C-peptide = 1.89 ng/ml on POD 3 (Fig. 5H). Swelling in the surgical area, observed in the early posttransplant period, subsided after drainage and antibiotic treatment. The recovery period was otherwise uneventful. Approximately 2 weeks after transplant, as the animal began to eat well, the EIRs were similar to pretransplant values. Fasting C-peptide levels decreased during the first 28 days after transplant, together with increases in the average values for FBG and PBG (Fig. 5H). Thereafter, from POD 28 to POD 53, average values for FBG (89.9 ± 42.5 mg/dl) and PBG (95.3 ± 39.1) decreased and were lower than those observed pretransplant (FBG: 117.1 ± 38.3; PPG: 139.1 ± 49.8) at comparable EIR. The potential contribution of the endogenous islet function at this stage is difficult to assess. An elective necropsy was performed on POD 55, after a meal-stimulated C-peptide value obtained on POD 46 (1.84 ng/ml) was similar to the one obtained pretransplant (2.21 ng/ml on POD −7). Histopathological analyses of sections of the bursa omentalis showed mild chronic inflammation and fibrosis (Fig. 5I, left), which suggested improved biocompatibility of EM CC compared to DM CC and abundant graft revascularization (Fig. 5I, right).

### CC syngeneic rat islets show improved in vitro functionality but reduced performance to naked syngeneic rat islets in the omental pouch of diabetic rats

**Fig. 6.**
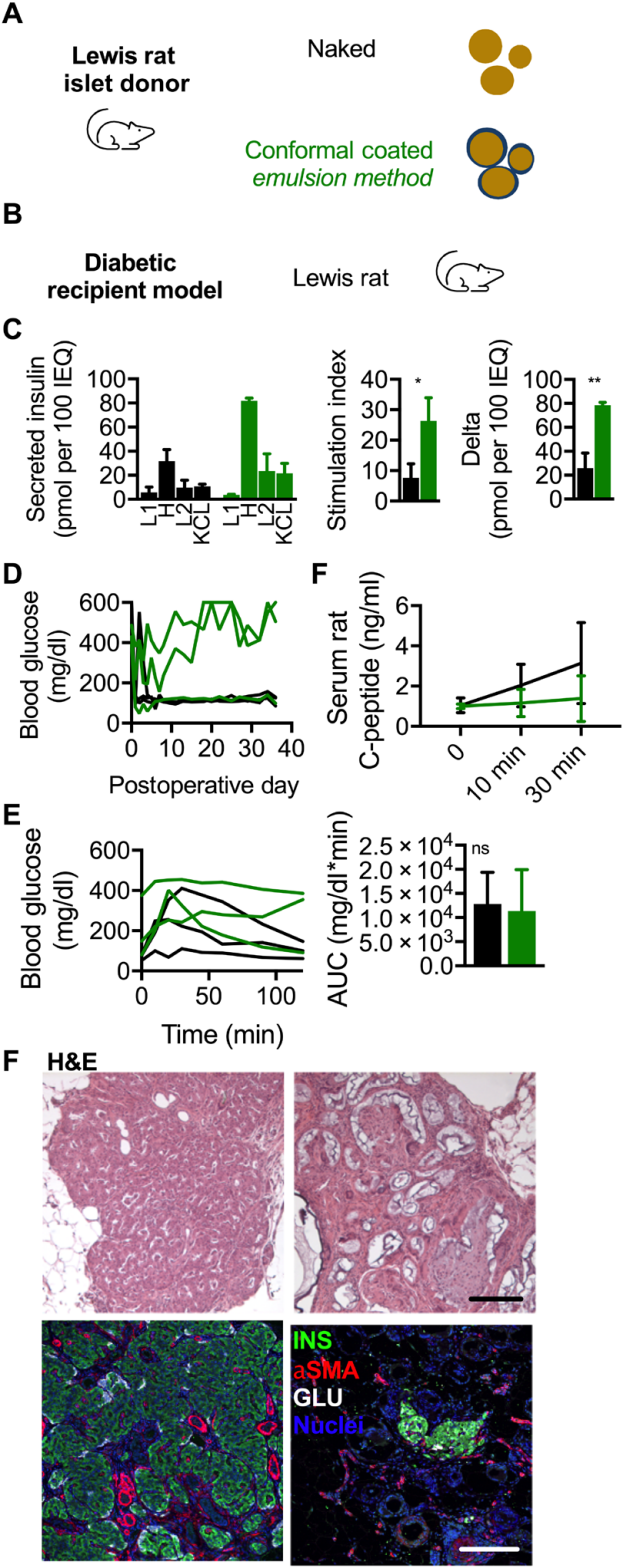
In vivo characterization of EM CC in syngeneic diabetic Lewis rat recipient model. (**A**) Schematic of Lewis rat islet donor and naked (black) and CC EM (green) islets. (**B**) Schematic of diabetic Lewis rat recipient models. (**C**) GSIS of naked (black) and CC EM (green) Lewis rat islets as absolute insulin (L1, 2.8 mM glucose; H, 20 mM glucose; L2, 2.8 mM glucose; KCl, 2.8 mM glucose and 30 mM KCl), stimulation index (H/L1), and delta (H-L1) (*n* = 3 technical repeats). *P < 0.05 and **P < 0.01. (**D**) Left: Daily blood glucose readings of rats receiving 1250 IEQ of naked (black, *n* = 3) and CC EM (green, *n* = 3) Lewis rat islets and (**E**) IPGTT blood glucose profile and calculated AUCs and (D, right) stimulated C-peptide measurement (*n* = 3) for both conditions. ns indicates *P* > 0.05. (**F**) Light microscopy images of H&E-stained (top row) and immunofluorescence confocal micrographs [bottom row; insulin (INS) in green, glucagon (GLU) in red, and nuclei in blue] of explanted omental tissue containing islet grafts from naked (left) and EM CC islets (right) (scale bars, 200 μm).

Rat islets from Lewis rat donors were conformal coated using the EM (Fig. 6, A and B). CC EM rat islets exhibited higher static GSIS functionality than naked islets (Fig. 6C, left) with respective stimulation indices (Fig. 6C, middle) of 7.6 ± 4.7 and 26.3 ± 7.6 (*P* = 0.02) and delta (Fig. 6C, right) of 25.9 ± 12.5 and 78.5 ± 2.4 pmol of insulin per 100 IEQ (*P* = 0.002). When transplanted with 1250 IEQ into the omentum of diabetic Lewis rats, naked and EM CC rat islet recipients exhibited a median reversal time of 1 and 6 days (*P* = 0.15), respectively, and mean postoperative blood glucose of 144 ± 88 and 325 ± 81 mg/dl (Fig. 6D, left; *P* < 0.0001), respectively. IPGTT was conducted and quantified by calculating the area under the curve (AUC) normalized to fasting blood glucose baseline for each group. The AUC (mean ± SEM) for naked islets and EM CC islets was 12,822 ± 3779 and 11,348 ± 4948 mg/dl*min (Fig. 6E, *P* = 0.82). In the rat host, one of three recipients of CC EM islets exhibited durable normoglycemia, whereas all the recipients of naked islets did so (Fig. 6D, left), suggesting lower in vivo functionality of CC EM rat islet syngeneic grafts than naked controls. C-peptide levels (Fig. 6D, right) increased for both naked and EM CC islet recipients between fasting and 30 min into IPGTT, though not in a statistically significant manner (two-way ANOVA, row factor, *P* = 0.18). C-peptide levels for naked and EM CC islet recipients at both fasting and 30 min into IPGTT were comparable (two-way ANOVA, column factor, *P* = 0.11). H&E-stained images revealed islet grafts with EM CC islets integrated into the host tissue (Fig. 6F, top row). Immunofluorescence staining revealed grafts with insulin- and glucagon-positive cells and α smooth muscle actin–positive vessels (Fig. 6F, bottom row). We concluded that while in vitro functionality of CC EM rat islets was comparable to naked controls, in vivo functionality in the omental pouch of syngeneic diabetic rat recipients was decreased.

### EM CCs are complete on MIN6 insulinoma cell clusters and showed functionality in spontaneously diabetic nonobese diabetic mice

**Fig. 7.**
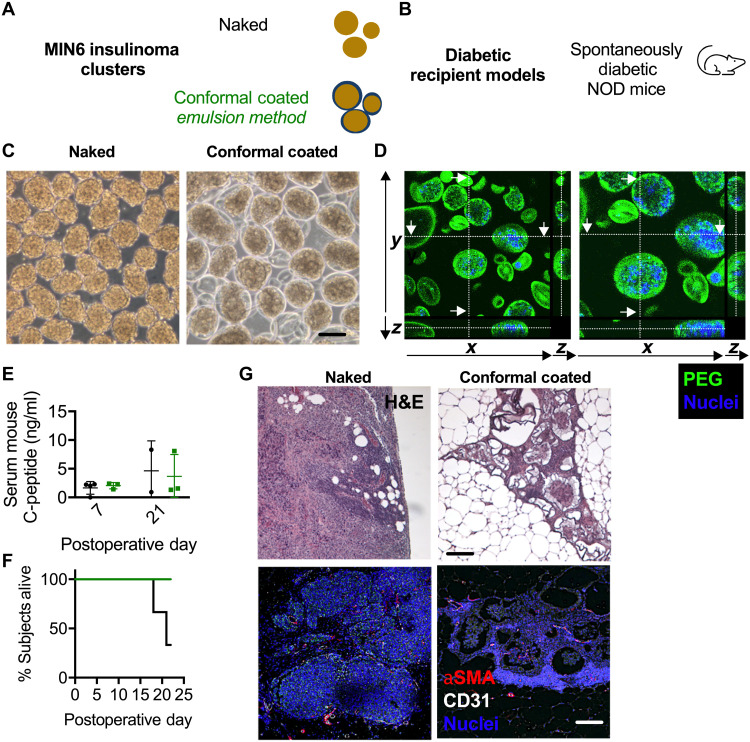
In vivo characterization of EM CC in allogeneic diabetic NOD mouse recipient model. (**A**) Schematic of naked (black) and CC EM (green) MIN6 insulinoma clusters. (**B**) Schematic of diabetic NOD mouse recipient models. (**C**) Phase-contrast image of naked (left) and CC EM MIN6 clusters (right; scale bar, 200 μm). (**D**) Confocal micrographs of CC EM MIN6 clusters showing nuclei (blue) and PEG EM CC (green) with projections in the XY, YZ, and XZ planes. (**E**) Serum C-peptide on POD 7 and 21 for NOD mice receiving 4000 IEQ of naked (black) and CC EM (green) MIN6 clusters. (**F**) Survival curve depicting diabetes reversal in NOD mice receiving naked (black) and CC EM (green) MIN6 clusters. (**G**) Light microscopy images of H&E-stained NOD fat pad grafts bearing naked (left) and CC EM (right) MIN6 clusters.

MIN6 insulinoma cell clusters with an average diameter of 143 μm were conformal coated using the EM (Fig. 7, A and B). Phase-contrast images (Fig. 7C) show naked and EM CC MIN6 clusters. Confocal micrographs of EM CC MIN6 clusters stained with Hoechst nuclear stain and anti-PEG antibody indicated that EM CCs were complete (Fig. 7D). Serum C-peptide of spontaneously diabetic nonobese diabetic mice (NOD) transplanted in the gonadal fat pad with either naked or EM-CC MIN6 clusters was measured at POD 7 and 21. While three of three recipients of CC clusters showed stable C-peptide and were alive until day 22, recipients of naked clusters expired at days 18, 21, and 22. Thus, CC MIN6 clusters improved survival of recipient diabetic NOD mice. H&E-stained images revealed MIN6 cluster grafts with EM CC clusters integrated into the host tissue (Fig. 7G) with immune infiltrates between but not within capsules, unlike naked MIN6 cluster grafts.

## DISCUSSION

While we were able to achieve high-quality CCs using our previously reported DM, we sought to improve the process by addressing three major concerns associated therein as identified from the NHP studies that we performed here for the purpose of translating this microencapsulation platform to humans with T1D. First, we augmented the process so that at no point would the islets be exposed to nonphysiological pH because this caused death of cells on the islet surface, which could elicit an immune response (*21*), as well as overall GSIS dysfunction, which was observed here on primary NHP islets because they are more sensitive to the process than primary islets from other animal sources and from humans. Second, we removed the peptide viscosity enhancer additive because it was reported to be immunogenic in its own right (*22*) and it contributed to the suboptimal biocompatibility that we observed after implantation in the NHP omentum. Third, we aimed at increasing the efficacy of the coating process, which is critical for translation to large numbers of human islets necessary for diabetes reversal in patients with T1D. We were able to implement changes to the CC process while preserving the hallmark “conformal coatings” of a covalently cross-linked PEG-based hydrogel on the islet surface. One of the major advantages of our CC technology is that the hydrogel capsule thickness is on the order of a few tens of micrometers (*16*). This contrasts with other microencapsulation technologies, where the overall diameter of the encapsulated islet is constant and can be quite large because techniques to generate microdroplets, such as electrostatic droplet generation, typically exhibit a minimum lower limit of droplet diameter that is still relatively large (*23*). It is important to recall that doubling the diameter of a sphere leads to an eightfold increase in volume and makes packing of particles less efficient. In practical terms, this means that the graft volume of CC islets would be much less than that of microencapsulated islets, and so they can be transplanted in confined and well-vascularized sites that are superior to the intraperitoneal space when considering proper metabolic control of coated islet recipients (*24*). We demonstrated this minimal graft volume by CC NHP islets—30,000 IEQ had an overall graft volume of approximately 1 ml, which could be easily accommodated in both the omental pouch (*25*) and the bursa omentalis (*26*, *27*). This is close to the graft volume of unencapsulated islets and close to 200 times less than that of microencapsulated islets (*28*).

Our previously reported CC DM used PEG-maleimide as the base polymer and PEG-dithiol as the cross-linker. The two materials combined to form a solid hydrogel via Michael-type addition reaction (*29*); the conversion of these two liquid components to a hydrogel occurs rapidly at physiological pH. The CC process requires that islets be resuspended in the aqueous hydrogel precursor (a mixture of the base PEG-maleimide polymer and the PEG-dithiol cross-linker) and subsequently run through the encapsulation device to form CCs around individual islets; it is appropriate for gelation to ensue only after capsule formation. Because of the rapidity of the Michael-type addition gelation reaction, it was therefore necessary to lower the pH of the hydrogel precursor to a pH of 3 to 3.5 to delay gelation until after capsule formation (*30*). Furthermore, we could only process 2000 IEQ of islets at a time with the DM because the coating process was limited by hydrogel gelation that would eventually ensue. We demonstrate here that this transient exposure to an acidic pH reduces the viability and insulin secretion of conformally coated NHP islets compared to naked NHP islet controls. To improve these outcomes, we modified our CC procedure so that a low pH was not necessary. Instead of resuspending the islets in the aqueous hydrogel precursor at pH 3.5, islets were resuspended in PEG-maleimide at physiological pH. The CC device was similarly used to produce CCs, after which the islets in the still-liquid phase coating were incubated in a water-in-oil emulsion containing the cross-linker dithiothreitol to permit gelation of the coating. Preventing any exposure of islets to acidic pH resulted in an overall improvement in CC islet viability and GSIS performance when compared to unencapsulated islets from the same preparation. Variability of NHP islet performance across different batches prevented a direct comparison between EM CC NHP islets and DM CC NHP islets from different batches, and we limited comparison to their own naked NHP controls. Because cross-linker was not applied until after encapsulation, we were able to coat 10,000 IEQ of islets at a time, a fivefold increase in throughput as compared to the DM. This improvement is critically important for translation of CC to humans, which requires encapsulation of several hundred thousand IEQ per patient with T1D. We studied the physical properties of the CCs and determined that those produced with the EM had similar elastic modulus (*31*) as well as diffusion properties (*32*) and permselectivity with IgG exclusion as compared to other fully cross-linked PEG hydrogels. As a result, we infer that the transfer of cross-linker from the emulsion to the still-liquid capsule is efficient and complete.

The CC process requires the aqueous phase (the liquid hydrogel precursor) to be of sufficiently high viscosity to enable complete and conformal capsule formation (*17*). This viscosity enhancement was previously achieved by adding either alginate (*18*), or Matrigel, or a self-assembling amphiphilic peptide derived from a human membrane protein and spider silk protein (VEP) (*22*). However, this peptide is immunogenic and was confirmed in our first NHP trial, and so we sought to remove it from the CC hydrogel platform. Instead of replacing the peptide with another distinct additive, partial cross-linking of the hydrogel base polymer was tested as a way to simplify the hydrogel platform while increasing the viscosity of the polymer as required by the CC process. We found that using a molar ratio of 5:3 of PEG-maleimide base polymer to PEG-dithiol cross-linker was sufficient to convert the watery base polymer to a flowable viscous liquid that was compatible with the CC device and allowed generation of CCs with pure PEG.

Given the improvements in in vitro outcomes using EM CC compared to their naked islet controls, we investigated the in vivo performance in immunocompromised NSG mouse in different transplant sites. First, better outcomes were achieved with a dose of 4000 IEQ as compared to 2000 IEQ, which is not surprising but highlights the dose-dependent nature of islet cell transplant outcomes. Second, the gonadal fat pad was a better transplant site than the intramuscular and subcutaneous spaces in terms of mean postoperative blood glucose levels, which is in agreement with what had been described previously (*33*). It was surprising that recipients in the subcutaneous and intramuscular sites exhibited higher (though not statistically significant) human (measured with a method that does not cross-react with mouse) C-peptide levels than recipients in the fat pad. We also observed that diabetes reversal takes longer to achieve using human islets when compared to mouse islets that we have used in previous studies (*18*), likely due to the large size of human islets affecting engraftment and survival, the lower sensitivity of mouse host to human insulin, the smaller fraction of β cells in human islets as compared to mouse islets (*34*), and the longer human donor organ cold ischemia time (*35*).

Last, in vivo performance of EM CC islets was dependent on the species of the recipient model. In the fat pad of the NSG mouse, we were able to achieve diabetes reversal. In rat, we could achieve good glucose tolerance during IPGTT, although diabetes reversal was only achieved in one of three recipients, indicating that the rat islet transplantation model is more challenging than murine islet transplantation models. In NHP, we demonstrated safety and feasibility of transplanting EM CC islets in a large-animal model and observed transient xenograft functionality (as increased C-peptide) and improved biocompatibility in the bursa omentalis. Of course, our experience transitioning from rodent to NHP recipient model highlights not only the difficulty of recapitulating promising mouse host findings in more complex models but also its importance as an intermediate step between rodent models and human trials. While we, like others (*20*), were not able to achieve insulin independence in the NHP, likely due to the limited efficacy of EM CC to provide immunoisolation to xenografts, we were able to demonstrate the practicality of transplanting CC islets in the omentum with an overall graft volume manyfold less than microencapsulated islets.

Testing immunoisolation of the EM CC platform was not the primary motivation of this work, because this was not a limitation observed with DM CC platform (*18*). However, our results indicate that EM CC hydrogels exhibit comparable physical properties than DM CC ones, including exclusion of IgG from coating hydrogels and improved survival of spontaneously diabetic NOD transplanted with DM CC MIN6 clusters, indicative of immunoisolation. As a next step, we will redouble our efforts to exploit synergies between encapsulation as a physical barrier and as a depot for delivery of local immunomodulation (*36*) to improve the survival of transplanted CC islets in an NHP immunologically mismatched allograft model.

Overall, we report the design and testing of an improved CC method for immunoisolation of cell clusters in clinically relevant transplant sites in different preclinical models of T1D. While the work is focused on T1D models, our EM CC platform is applicable to other cell cluster types for application in other regenerative medicine applications where transplantation of allogeneic cells could be beneficial and where limited volume and physiological nondelayed trophic factor secretion is a main concern. Furthermore, our studies comparing outcomes of the EM CC platform in different preclinical models with variable results depending on the specific model and the transplantation sites is critical knowledge for cell transplantation research in T1D and other fields.

## MATERIALS AND METHODS

### Study design

The overall aim of this study was to improve on our original CC method so as to increase islet cell viability and insulin-secreting function. This new method was enabled via the development of a cross-linking emulsion that allowed hydrogel cross-linking after capsule formation so that a harmfully acidic polymer pH was not required to delay hydrogel cross-linking. Islets of Langerhans from various donor species (mouse, rat, NHP, and human) were conformally coated with the EM. These coated islets were transplanted in a variety of diabetic recipient contexts: different graft sites and doses in immunocompromised mouse, immunocompetent rat host, immunocompetent islet reactive mouse host, and immunocompetent NHP host. Studies in these several models allowed a broad inquiry of the performance of conformally coated islets in vivo. All animal studies were performed under protocols reviewed and approved by the University of Miami Institutional Animal Care and Use Committee.

### In vitro static GSIS, phase-contrast imaging, and CC thickness measurement

Islet functionality was assessed through static GSIS assay in 24-well plate-cell culture inserts (EMD Millipore, Temecula, CA) at a density of 250 IEQ per well in triplicate wells by sequential exposure with 2.8 mM glucose (L) for 1 hour, 20 mM glucose (H) for 1 hour, 2.8 mM glucose (L) for 1 hour, and 30 mM KCl for 1 hour. Phase-contrast images were acquired using a Leica DM IL inverted microscope. Dithizone-stained phase-contrast images were acquired by staining islets in saturated dithizone solution (Millipore Sigma, St. Louis, MO) in 20% dimethyl sulfoxide in HBSS^−/−^ (no Ca^2+^ or Mg^2+^) for 5 min. Coating thickness was measured using ImageJ software [National Institutes of Health (NIH), Bethesda, MD].

### In vitro dynamic perifusion and DNA quantification

To assess the insulin release kinetics of islets with greater temporal resolution, islets were perifused with solutions of different glucose concentrations and the released insulin was measured. A Peri4-02 perifusion machine (Biorep Technologies, Miami, FL) was used to conduct this assay as previously reported. Briefly, 100 IEQ of islets per replicate for each condition of interest were loaded into a Perspex microcolumn in a fashion where they were sandwiched between layers of a microbead slurry Bio-Gel P-4 (Bio-Rad Laboratories, Hercules, CA). Solutions of glucose or KCl in a Krebs-Ringer buffer base were perifused around islets destined for mouse or rat recipients in the following order: L (low; 3 mM glucose) for 1 hour as preconditioning, L for 8 min, H (high; 11 mM) for 20 min, L for 15 min, KCl (30 mM KCl, 3 mM glucose) for 10 min, and L for 8 min. Solutions of glucose or KCl in a Krebs-Ringer buffer base were perifused around islets destined for NHP recipients in the following order: L (3 mM glucose) for 1 hour as preconditioning, L for 5 min, H (11 mM) for 10 min, L for 15 min, KCl (25 mM KCl, no glucose) for 5 min, and L for 5 min. Perifusion solutions were maintained at 37°C and flowed at a rate of 100 μl/min. Samples of 100 μl were collected each minute and immediately cooled by the perifusion machine to 4°C. Insulin concentration in each sample was assessed by enzyme-linked immunosorbent assay (ELISA): For samples from NHP or human islets, a Human Ultrasensitive Insulin ELISA kit (Mercodia, Uppsala, Sweden) was used, and for mouse or rat islets, a Mouse or Rat Insulin ELISA kit (Mercodia, Uppsala, Sweden) was used. After perifusion, islets in Bio-Gel P-4 slurry were collected in 1 ml of T-PER Tissue Protein Extraction Reagent, vortexed, and frozen. To quantify DNA, samples were thawed and the Quant-iT PicoGreen Double-Stranded DNA Assay Kit (Thermo Fisher Scientific, Waltham, MA) was used.

### Viability assessment through live/dead staining and confocal imaging

Islets were stained in culture medium for 1 hour with the following added: calcein AM and ethidium homodimer (1:1000 dilution for both) from the Live Dead Viability/Cytotoxicity Kit (Thermo Fisher Scientific, Waltham, MA) and Invitrogen Hoechst (1:2000 dilution; Thermo Fisher Scientific, Waltham, MA). After staining, islets were washed with HBSS^−/−^ thrice. Islets were imaged in 3D in 1-μm-thick z-stacks using an SP5 confocal microscope (Leica Microsystems, Wetzlar, Germany) in a Lab-Tek chambered slide (Thermo Fisher Scientific, Waltham, MA). ImageJ software (NIH, Bethesda, MD) was used to quantity select live/dead images to determine the viability.

### CC encapsulation

#### Direct method

This article is the first instance where we refer to our CC platform reported previously (*17*–*19*) as CC DM; no distinction was required in the past because it was the only method. CC DM was conducted as described previously (*17*–*19*).

#### Emulsion method

We refer to the method we report here as CC EM. The EM was conducted similar to the DM described above, with the following modifications. First, no VEP, Matrigel, self-assembling amphiphilic peptide (PepGel) additive, or self-assembling amphiphilic PEG–oligoethylene sulfide nanofibers were added to the base polymer, which was custom-synthesized 5% (w/v), 10 kDa, 8-arm, 75% functionalized PEG-maleimide (JenKem, Plano, TX). The viscosity of this base polymer aqueous solution was increased by partial cross-linking of 15% with 2-kDa PEG-dithiol (JenKem, Plano, TX) in a 5:3 molar ratio (base:cross-linker). This was achieved by first lowering the pH of the base polymer to three and then adding the cross-linker in a 42.9% (w/v) solution. The pH must be lowered with 1 N HCl (Millipore Sigma, St. Louis, MO) to allow even mixing of the cross-linker and prevent instantaneous and heterogeneous gelation. The pH was then made neutral by adding small amounts of 1 N NaOH (Millipore Sigma, St. Louis, MO). Islets were resuspended in the neutral pH, partially cross-linked base polymer at a density of 100,000 IEQ/ml and ran through the CC device as described previously (*17*–*19*). Instead of a 50-ml conical tube with 10 ml of PPG/10% Span80, the islets were collected in a 150-ml glass beaker. As implied in the name, the EM uses an emulsion to engender gelation of the nascent liquid base polymer CCs on the islets. To achieve this, the emulsion was dispensed on the exterior of the glass capillary of the CC device so that it met the CC islets in PPG/10% Span80 at the exit orifice of the capillary. The emulsion was dispensed at a rate of 8 ml/min. The emulsion was prepared as follows: to a 1-liter beaker with a large almond shaped stir bar with 500 ml of PPG/10% Span80 stirring, 33 ml of 25 mg/ml of molecular biology–grade dithiothreitol (VWR, Radnor, PA) in HBSS^−/−^ was added drop-wise. After collection in the beaker, the CC islets were incubated in the beaker for 12 min. To purify the coated islets from the emulsion, the suspension was poured into a 1-liter beaker with 200 ml of light mineral oil (Millipore Sigma, St. Louis, MO) stirring at 240 rpm. Then, the purification beaker was top-offed to a total volume of 500 ml with HBSS^−/−^ and stirred for 2 min. The coated islets were then collected by centrifugation at 430 relative centrifugal field and washed in HBSS^−/−^ several times before culturing in medium.

### Fluorescence recovery after photobleaching

FRAP was used to quantify the diffusion coefficient within the polymer capsule. FRAP measurements were performed at the central plan of the encapsulated beads. This measurement location was established by imaging a 3D z-stack of the encapsulated beads preceding the FRAP experimentation. Three different areas to be bleached were selected within the polymer capsule between the bead and the surrounding fluid. Each area was a circle of 5- to 20-μm diameter (determined by the thickness of the capsule). The three regions were bleached for a total of 10 to 15 s using an argon laser (488 nm) set to full power (65 mW). Immediately after bleaching, time-lapse images of the full field of view were captured each second for at least 3 min. Changes in fluorescence intensity stemming from the diffusion of the fluorescently labeled molecule were monitored in each of the three regions. Custom Matlab software was used to fit the Soumpasis equation to the data

$$f(t)=e^{-2\frac{\tau_{D}}{t}}[I_{0}(\frac{2\tau_{D}}{t})+I_{1}(\frac{2\tau_{D}}{t})]$$

where *I*_0_ and *I*_1_ are modified Bessel functions and *t*_D_ is the recovery constant. The custom Matlab software outputted *t*_D_, which was then used to calculate the diffusion coefficient, *D*, after accounting for the diameter of the bleached region, r

D=0.224r2τ12

The intensity profile across the entire encapsulated bead was used to determine the partition coefficient. Using custom Matlab code, we found the average fluorescent intensity inside the capsule (*I*_capsule_) and the average fluorescent intensity outside the capsule (*I*_media_). The ratio of these values was defined as the partition coefficient, *P*

$$P=\frac{I_{\text{capsule}}}{I_{\text{media}}}$$

#### Atomic force microscopy

Young’s modulus of elasticity of encapsulated beads was measured using a custom-built AFM that has been described in detail previously (*37*–*39*). Measurements were performed by indenting the samples with a silicon nitride triangle cantilever with a 2-μm silica-bead affixed to the apex (*k* = 0.1 N/m; Novascan Technologies Inc., USA). A piezoelectric mechanism (60-μm maximal expansion; P-841.40, Physik Instrumente, Germany) was programmed to lower the cantilever onto the sample vertically at the rate of 15 m/s. Upon reaching the maximum indentation force of 1 V (corresponding to ~15 nN), the cantilever was retracted. According to the principle of AFM indentation, the cantilever will undergo a combination of bending and indentation of the sample during interaction. The cantilever bending is monitored and recorded with the reflection of a laser from the back of the cantilever to a position-sensitive photodiode. Measurements were repeated 10 times at three different locations around the central region of five different encapsulated beads. Young’s modulus of elasticity was calculated from each measurement using custom-developed Matlab software. The photodiode voltage versus piezoelectric displacement recorded during the measurement scans was converted to force versus indentation after accounting for the cantilever spring constant (0.12 N/m) and the cantilever bending response on hard surface, assuming no indentation. The force versus indentation curves were analyzed using the Hertz model for a spherical indenter

F=4ER3(1−ν2) D32

where *F* is the measured force (N), *E* is the Young’s modulus of elasticity (Pa), ν is the Poisson’s ratio (0.49), and *D* is the measured indentation (m). Each curve fit was verified visually. Raw data were analyzed using the interquartile range method to identify and exclude outliers. Using this method, any value 1.5 times above the third quartile or 1.5 times below the first quartile were excluded as outliers. Because at least 10 repeat measures were acquired per measurement location, we applied this outlier analysis method to the repeat measures for each location. If a point was determined to be an outlier in accordance with the interquartile range method, it was excluded from the average value for that location. Then, the same process was applied to the average values for all three locations for each group, and any outliers were excluded from the averages for each sample.

#### Islet isolation

Rat pancreatic islets were isolated and cultured as described previously (*17*). Male Lewis rats (250 g) were purchased from Charles River Laboratories (strain code: 004) and used as islet donors.

Human islets were purchased from Prodo Laboratories Inc. (Aliso Viejo, CA). NHP islets were isolated as described previously (*40*).

#### Min6 cluster formation

Min6 cells were purchased from AddexBio and cultured in a T25 flask. At 60 to 70% confluency, they were passaged to a T75 flask. The insulinoma cells were cultured in Dulbecco’s Modified Eagle’s Medium (DMEM) supplemented with 15% fetal bovine serum, 0.05 mM 2-mercaptoethanol, 30 mM glucose, and 1% penicillin/streptomycin. Cells were counted using trypan blue exclusion assay. Min6 cells were seeded at a density of 1 million cells/ml in a 30-ml spinner flask (ABLE Biott, distributed by Reprocell, Beltsville, MD) stirring at 70 rpm. Clusters formed as early as day 2 and were used on day 4 of suspension culture. The medium was replaced every 2 days by letting the clusters settle for 5 min under the biosafety cabinet and subsequently aspirating the media and replacing it with fresh media up to 30 ml of final volume. Successful cluster formation was determined by light microscope pictures, single-cell viability, and glucose-stimulated insulin response.

#### Islet recipient mice

Male NOD.Cg-PrkdcscidIl2rgtm1Wjl/SzJ (hereafter NSG male) were purchased from The Jackson Laboratory (Bar Harbor, ME). Animals were 5 weeks of age and between 20 and 26 g for males and 15 and 22 g for females when received.

Diabetes was induced at 7 weeks via multiple sequential low doses of streptozotocin (Sigma-Aldrich, St. Louis, MO). Briefly, a working solution of streptozotocin was prepared in citrate buffer at a pH of 4.5 and intraperitoneally injected at a dose of 40 mg/kg for NSG males, 5 days in a row. Animals were not fasted before streptozotocin administration. Animals received up to 2 ml of saline intraperitoneally daily in the period between diabetes induction and transplantation. Mice were considered diabetic after three consecutive blood glucose readings greater than 250 mg/dl.

Female and male NOD (stock no. 001976) and T cell receptor transgenic CD8^+^ T cell NOD.NY8.3 (stock no. 005868) were purchased from The Jackson Laboratory (Bar Harbor, ME) and bred in house. Mice that developed diabetes (as three consecutive blood glucose readings >250 mg/dl) at age 14 to 25 weeks were used in this study. The recipients were equally divided among the two treatment groups: naked and CC MIN6 clusters.

#### Islet recipient rats

Female Lewis rats (up to 200 g) were purchased from Charles River Laboratories (strain code: 004) and used as islet transplant recipients.

#### Islet transplantation in mice and rats

For the gonadal fat pad graft site in mice, the abdomen of recipient mice was shaved, and the animals were subsequently anesthetized with 2% isoflurane inhalant. The abdomen was sanitized with an alcohol towelette. A midline incision approximately 1.5 cm long was made through the skin and linea alba on the mouse abdomen. The gonadal (epididymal or mammary) fat pads were located, exposed, and unfurled as a flat plane supported by saline-moistened gauze. Islets were resuspended in 10 μl of citrated autologous plasma (obtained just before transplant from healthy, gender-matched, and strain-matched donors) and deposited on the fat pad (half of islets on each fat pad; as much as possible in tracts along the major blood vessels of the fat pad). Five microliters of recombinant human thrombin (1000 U/ml; Recothrom, ZymoGenetics, Seattle, WA) was applied on each fat pad to engender plasma clotting so as to immobilize the islets. Each fat pad was then refolded and glued with 15 μl of autologous plasma and 5 μl of recombinant thrombin. The folded fat pads were then resituated in the abdominal cavity. The abdominal muscle was sutured, and the skin was stapled.For the intramuscular graft site in mice, the two hindlimbs of the recipient mouse were shaved. A small incision about 0.25 cm was made near the skin superficial to the knee joint. Islets were resuspended in 10 μl of autologous plasma and withdrawn into a 24-gauge intravenous catheter (Terumo, Tokyo, Japan). The total mass of islets to be transplanted was delivered in four separate injections: on each hindlimb, a fourth of the islet mass in the muscles forming the quadriceps in two tracts per leg. The catheter was inserted into the muscle ~1 cm, and the intended dose of islets was delivered as the catheter was withdrawn to create a linear tract of islets. The incision was stapled closed.For the subcutaneous graft site in mice, the transplant procedure was conducted as described previously (*41*), where the overall graft was divided in half between each side of the abdomen. Briefly, a 0.25-cm incision was made in the inguinal area of the mouse abdomen. The inferior epigastric artery was identified, and a small pocket was made superior to it to expose the inguinal subcutaneous white adipose tissue. Islets were resuspended in 10 μl of autologous plasma and were deposited in the pocket, and 5 μl of recombinant thrombin was added on top. The skin incision was stapled.For the omental pouch in rats after laparotomy, the omentum was exposed; islets were resuspended in 10 μl of autologous plasma (obtained just before transplant from healthy, gender-matched, and strain-matched donors) and deposited on the center of the omentum. Five microliters of recombinant human thrombin (Recothrom) was applied to immobilize the islets. The omentum was folded on itself or facing the stomach wall and/or native pancreas and glued with 15 μl of autologous plasma and 5 μl of recombinant thrombin. The abdominal wall and skin are sutured.

#### Blood glucose monitoring

Daily blood glucose measurements were performed on blood from tail vein prick using a Contour Next blood glucose monitor (Bayer, Leverkusen, Germany). Diabetes reversal was considered as three consecutive blood glucose readings below 250 mg/dl.

#### IPGTT, random, fasting, and stimulated C-peptide

A 30-μl blood sample (random C-peptide) was collected via submandibular vein prick for mouse recipients and tail vein prick for rat recipients and assayed for serum human C-peptide using a Stellux Chemiluminescence Human C Peptide ELISA kit (ALPCO, Salem, NH) for mice receiving human islets and with Mouse C Peptide ELISA (ALPCO, Salem, NH) for mice receiving MIN6 clusters and with Rat C Peptide ELISA for rats receiving rat islets. An IPGTT was conducted as follows: Mice or rats were fasted overnight before IPGTT, and then a 30-μl blood sample was collected via submandibular vein prick for mice or tail vein prick for rats just before IPGTT. Mice or rats were then intraperitoneally injected with 2 g per kilogram of glucose. Blood glucose measurements were taken via tail vein at time 0, 10, 20, 30, 45, 60, 90, and 120 min after administration. A 30-μl blood sample was collected at 0 min (before glucose administration) and 30 min after glucose administration via submandibular vein prick (stimulated C-peptide). Blood samples were assayed for C-peptide as described above.

#### NHP recipients

Donor and recipient male Mauritian cynomolgus monkeys were obtained from the Mannheimer Foundation (Homestead, FL) and were specific pathogen–free. Diabetes (fasting C-peptide levels <0.3 ng/ml) was induced by administration of streptozotocin at 100 mg/kg as previously reported (*40*) and verified 4 weeks later via a meal-stimulated C-peptide (<0.5 ng/ml). Blood glucose levels were monitored two to three times daily via heel stick. Subcutaneous insulin (Humulin R, Lilly, or Humulin R plus Lantus, Sanofi) was individually tailored to target blood glucose levels of 100 to 150 mg/dl before and after transplantation. Fasting plasma C-peptide levels were obtained after an overnight fasting and were assessed by electrochemiluminescence immunoassay using a Cobas analyzer (Roche Diagnostics Inc.). Daily EIR, weekly fasting C-peptide, and monthly hemoglobin A1c were assessed.

##### Conformal coating—direct method

Under the cover of clinically applicable immunosuppression, a diabetic cynomolgus monkey (H15C35) was transplanted with a marginal mass of ABO-compatible major histocompatibility complex–mismatched, CC DM allogeneic islets (5000 IEQ/kg). Using laparoscopic surgery, the CC islets were transplanted within a biologic scaffold in an omental pouch. The suspension of CC islets in recipient’s plasma was first delivered onto the omentum, followed by delivery of recombinant human thrombin (Recothrom) to induce gelation of the biologic scaffold. After adding additional recipient’s plasma to quench the remaining thrombin, the omentum was pulled on top of the gelled islets forming a pouch, and the edges of the pouch were sealed with additional thrombin. Induction therapy consisted of basiliximab at 0.3 mg/kg intravenously on POD 0 and 3 plus anti-inflammatory treatment with Enbrel (etanercept) at 0.8 mg/kg intravenously on POD 0 and 0.4 mg/kg subcutaneously on POD 3, 7, and 10. Maintenance therapy included daily FK506 (tacrolimus) and rapamycin (to achieve trough levels of 4 to 6 and 12 to 20 ng/ml, respectively). Trough levels for FK506 and rapamycin were measured weekly.

##### Conformal coating—emulsion method

A diabetic cynomolgus monkey (H17C59) was used as recipient of CC EM human islets under the cover of costimulatory blockade. Orencia (CTLA4-Ig; 20 mg/kg intravenously) was administered on POD −1, 0, 3, 7, 14, 21, and 28 and weekly thereafter and anti-CD154 ([5C8H1], NHPRR, 20 mg/kg intravenously) on POD −1, 0, 3, 7, 14, 21, and 28 and every 10 days thereafter. Using laparotomy, on POD 0, 20,358 IEQ/kg suspended in Hanks containing 2% recipient’s heat-inactivated serum was delivered into the bursa omentalis.

### Anti-PEG staining to assess coating completeness

PEG staining was performed by blocking the samples in HBSS^+/+^ (Gibco, Massachusetts, USA) supplemented with 2% bovine serum albumin (BSA; Sigma-Aldrich, St. Louis MO), 10% chicken serum (Sigma-Aldrich, St. Louis, MO), and 0.2% Triton (EMD, Burlington, MA) for 30 min at room temperature. Subsequently, the samples were incubated in anti-PEG antibody solution (Abcam, Cambridge, MA; 1:500) diluted in 2% BSA in HBSS^+/+^ for 1 hour at room temperature. The samples were washed with HBSS^+/+^ and incubated with SA-488 secondary antibody (1:200) diluted in 2% BSA in HBSS^+/+^ for 1 hour protected from light. Samples were washed again with HBSS^+/+^ followed by a nuclear counterstain with Hoechst (1:1000) in HBSS^+/+^ for 30 min protected from light. Samples were washed again with HBSS^+/+^ and placed in a glass-bottom petri dish and imaged using SP5 Leica inverted confocal microscope.

### Statistical analysis

Data are presented as means ± SD unless otherwise noted. Statistical significance was determined using GraphPad Prism (GraphPad Software, La Jolla, CA) via either two-tailed Student’s *t* test or one-way ANOVA, followed by Tukey’s post hoc test. Survival data were analyzed using a log-rank Mantel-Cox test. A *P* value of less than 0.05 was considered significant.

## Notes

View/request a protocol for this paper from *Bio-protocol*.
