# Supplementary material for: Performance of islets of Langerhans conformally coated via an emulsion cross-linking method in diabetic rodents and nonhuman primates
Source: Sci Adv. 2022 Jun 29;8(26):eabm3145. doi: 10.1126/sciadv.abm3145 (PMC9242596; doi:10.1126/sciadv.abm3145)
Supplement: Supplementary file 1 — Tables S1 and S2 [file sciadv.abm3145_sm.pdf]

Science Advances
AAAS

Supplementary Materials for

Performance of islets of Langerhans conformally coated via an emulsion cross-linking method in diabetic rodents and nonhuman primates

Aaron A. Stock *et al.*

Corresponding author: Alice A. Tomei, atomei@miami.edu

Sci. Adv. **8**, eabm3145 (2022)
DOI: 10.1126/sciadv.abm3145

This PDF file includes:

Tables S1 and S2

SUPPLEMENTARY INFORMATION

Table S1—Human Islet Donor Data

**Donor**	**1**	**2**	**3**	**4**
Age	38 y	37 y	51 y	44 y
Sex	Male	Male	Female	Female
Ethnicity	Hispanic	Caucasian	Caucasian	Hispanic
BMI	27.8	23.9	24.8	24.1
HbA1c	5.4%	5.8%	5.2%	5.8%
Cause of Death	Head trauma	Head trauma	Stroke	Aneurysm
IEQ per Islet	1.08	1.25	1.4	1.1

Table S2—Conformal Coating Yield (Human Islets)

	Emulsion Method	Direct Method
Number of Donors/Encapsulations	3	6
Number of IEQ/run	5,000-10,000	2,000
Encapsulation Yield (%) of Islets	61.3±8.1	57±16.2
